# Supplementary material for: Anatomically matched spatial comparisons identify Trps1 as a regulator of knee interzone formation
Source: bioRxiv. 2026 Oct 30:2026.10.28.762640. Preprint. [Version 1] doi: 10.64898/2026.10.28.762640 (PMC13734064; doi:10.64898/2026.10.28.762640)

Supplementary 1

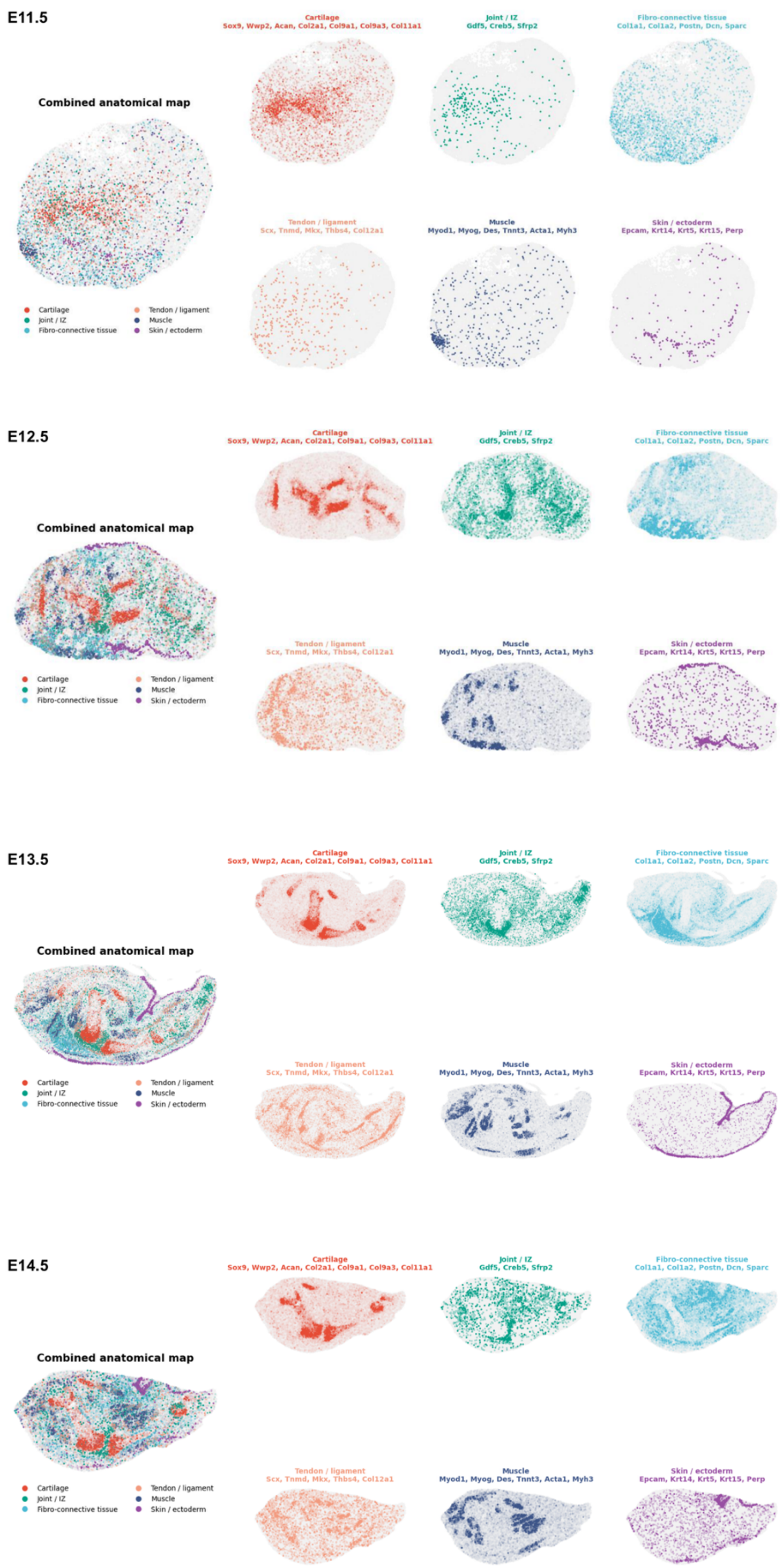

Supplementary 2

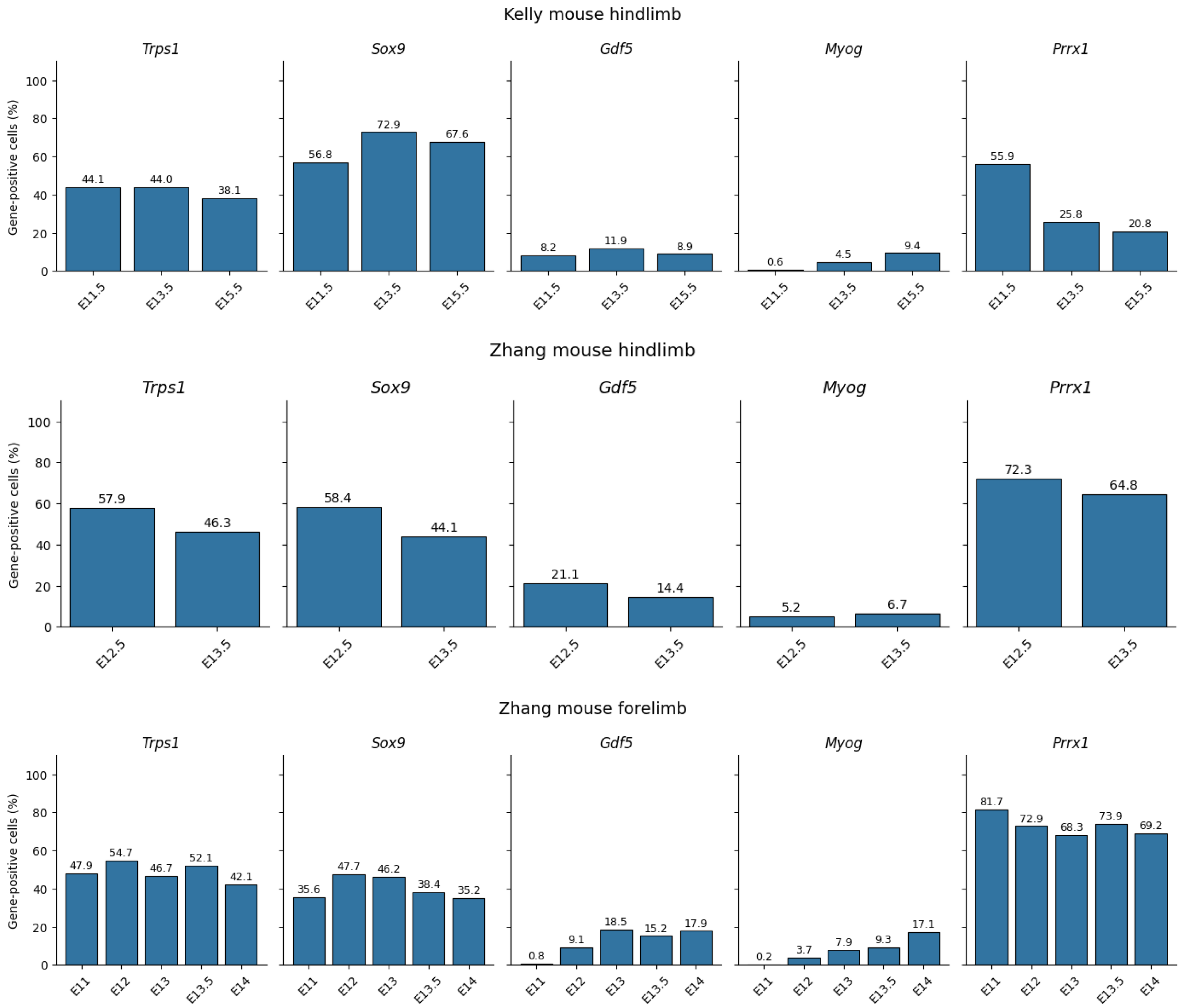

Supplementary 3

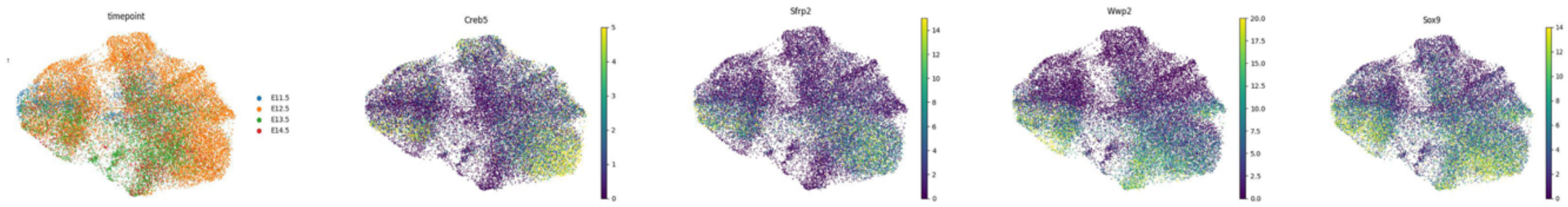

Supplementary 4

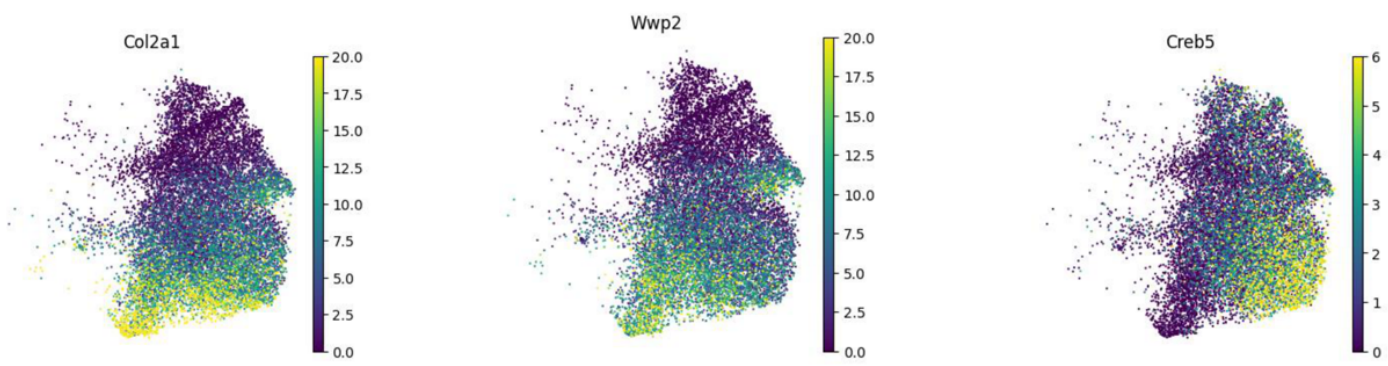

Supplementary 5

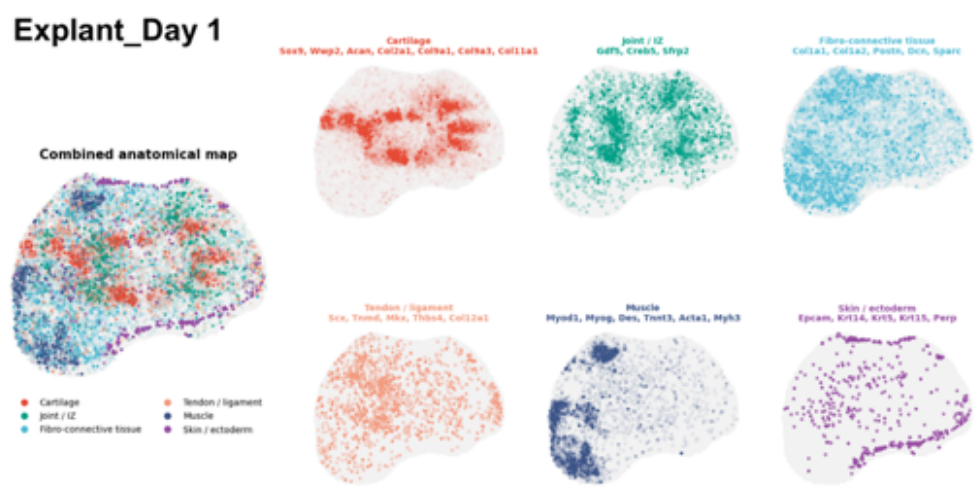

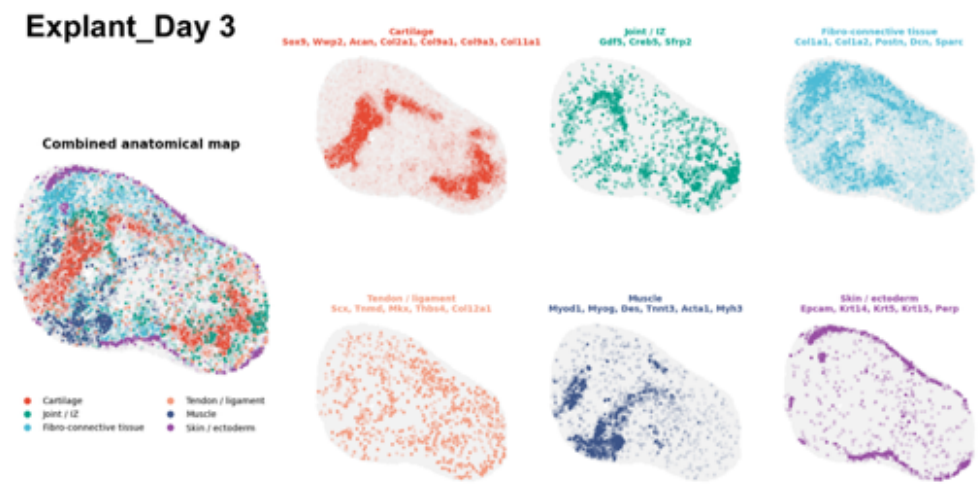

Supplementary 6

siTrps1:s211985

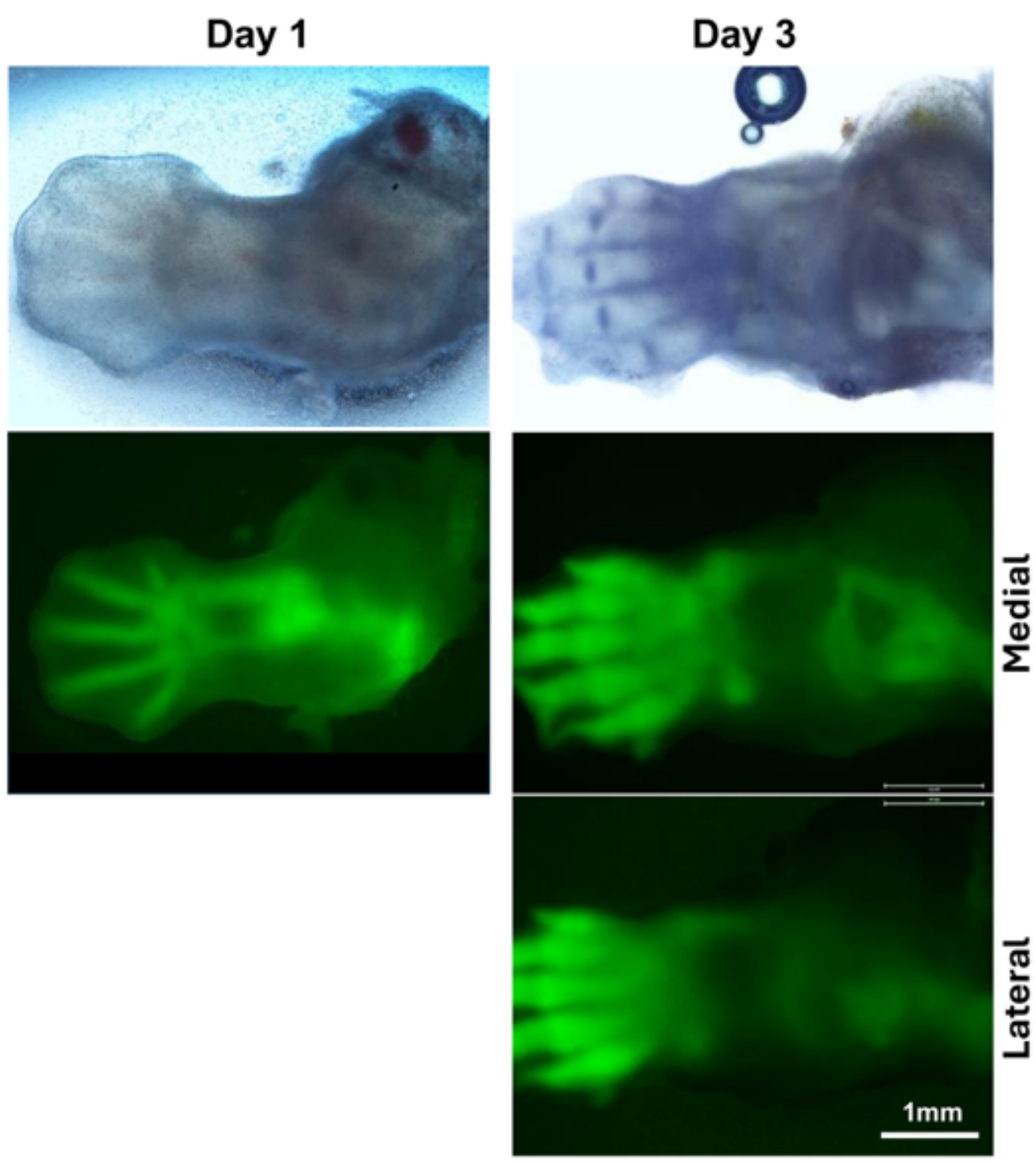

Supplementary 7

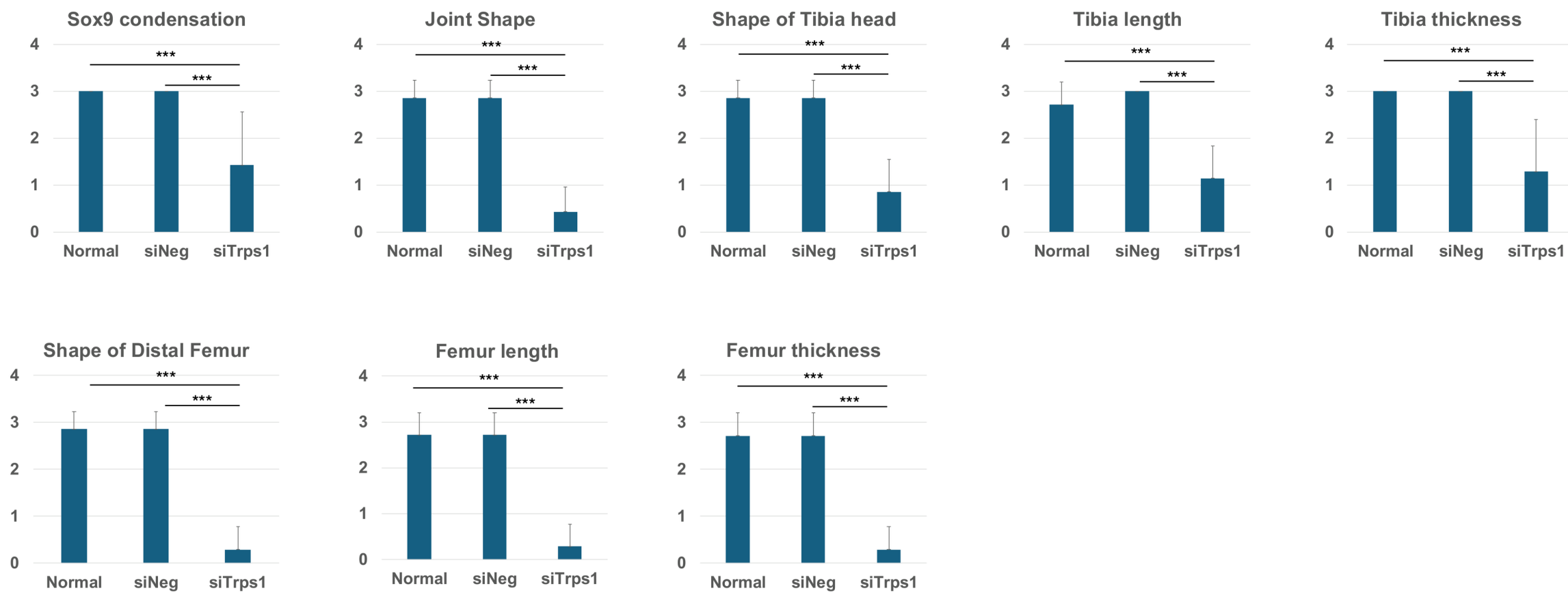

Supplementary 8

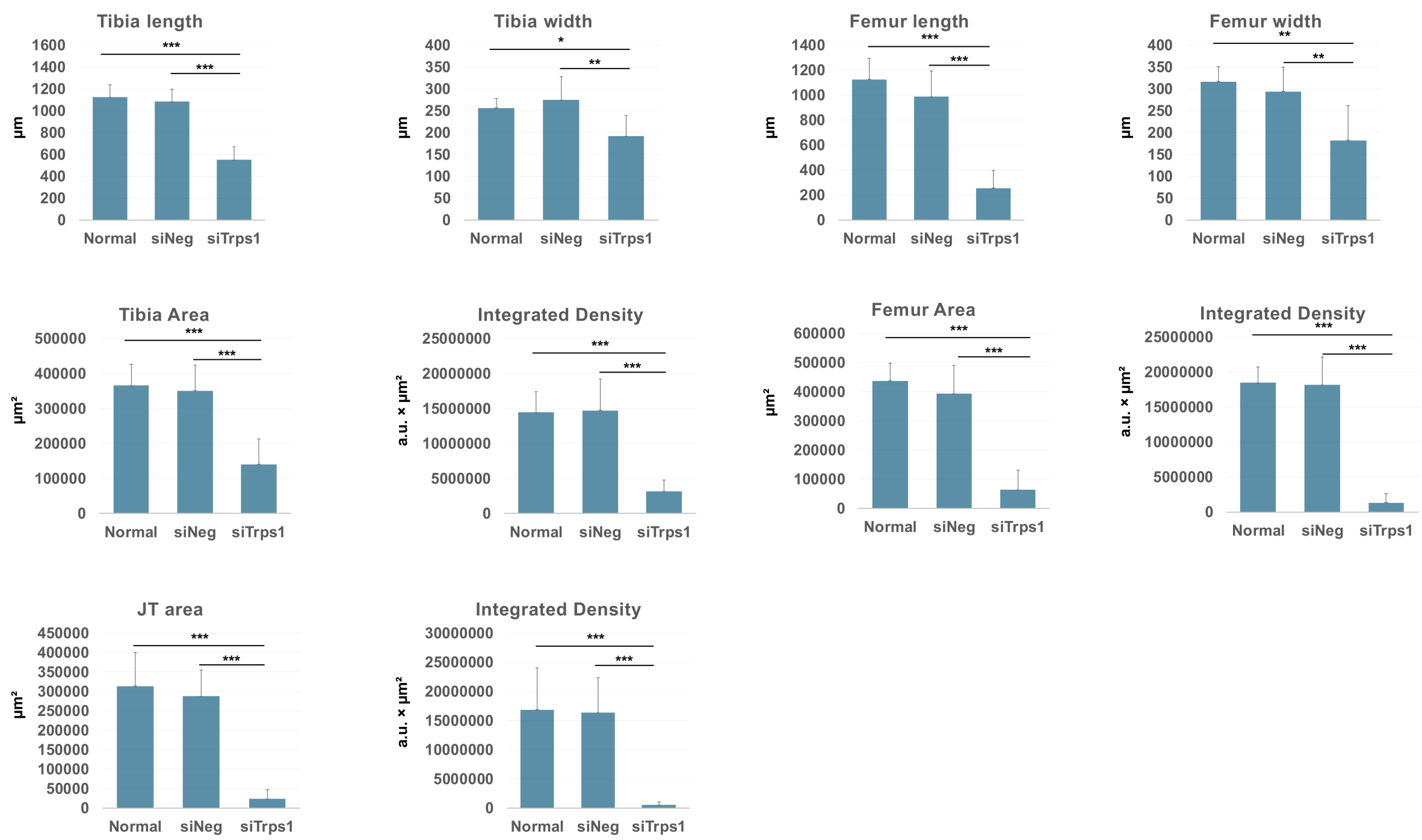

Supplementary 9

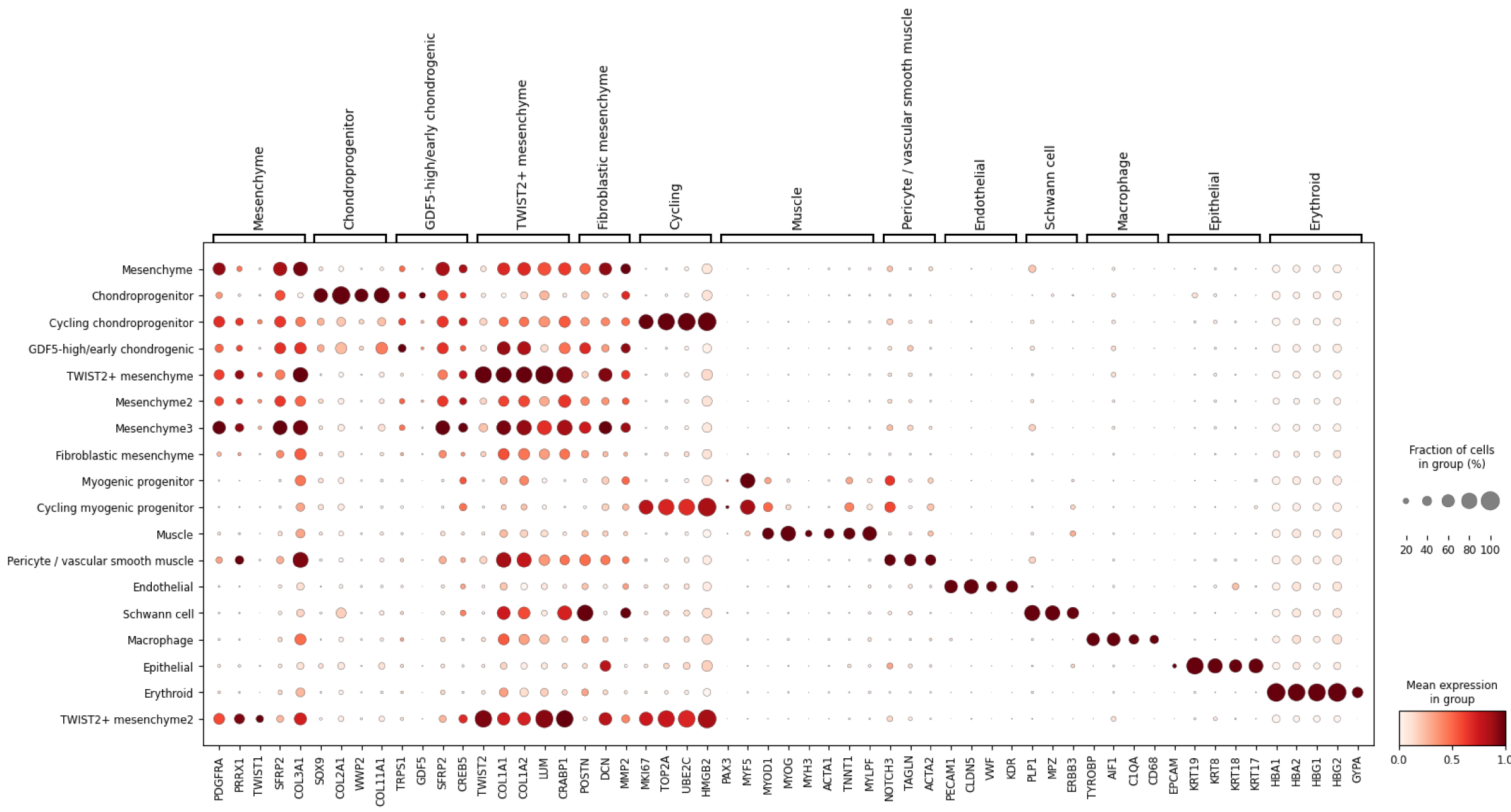

Supplementary 10

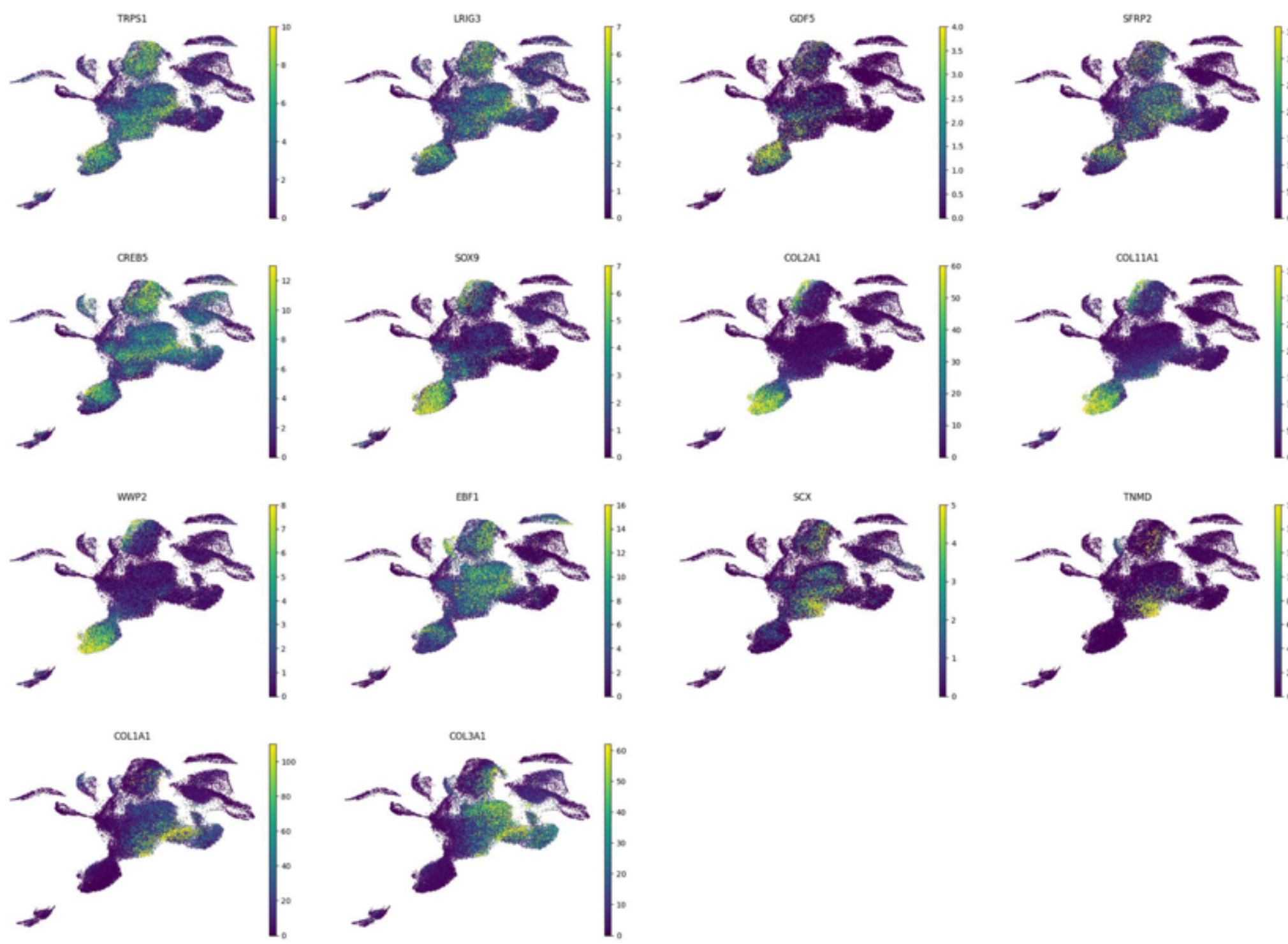

Supplementary 11

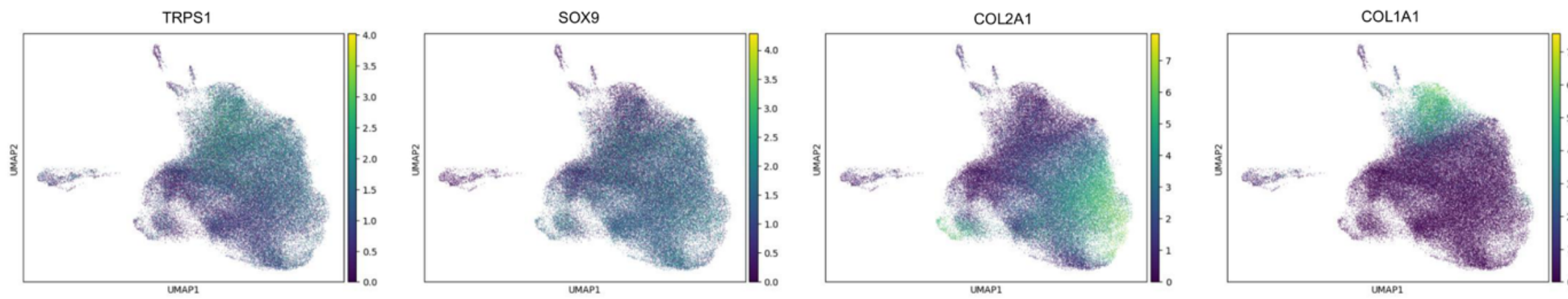

Supplement: Supplementary file 1 [file media-1.pdf]
